# Reduced Prenatal Pulmonary Lymphatic Function Is Observed in *Clp1*^*K/K*^ Embryos With Impaired Motor Functions Including Fetal Breathing Movements in Preparation of the Developing Lung for Inflation at Birth

**DOI:** 10.3389/fbioe.2020.00136

**Published:** 2020-03-06

**Authors:** Kitti Szoták-Ajtay, Dániel Szõke, Gábor Kovács, Judit Andréka, Gábor B. Brenner, Zoltán Giricz, Josef Penninger, Mark L. Kahn, Zoltán Jakus

**Affiliations:** ^1^Department of Physiology, Semmelweis University School of Medicine, Budapest, Hungary; ^2^MTA-SE “Lendület” Lymphatic Physiology Research Group of the Hungarian Academy of Sciences and the Semmelweis University, Budapest, Hungary; ^3^Department of Pharmacology and Pharmacotherapy, Semmelweis University School of Medicine, Budapest, Hungary; ^4^Institute of Molecular Biotechnology of the Austrian Academy of Sciences, Vienna, Austria; ^5^Department of Medical Genetics, Life Science Institute, University of British Columbia, Vancouver, BC, Canada; ^6^Perelman School of Medicine, University of Pennsylvania, Philadelphia, PA, United States

**Keywords:** fetal breathing movements, pulmonary lymphatics, lung development, genetic mouse models, organ-specific lymphatic function

## Abstract

Embryonic lungs must be inflated immediately after birth to establish respiration. In addition to pulmonary surfactant, recently, we have revealed lymphatic function as a previously unknown regulator of prenatal lung compliance that prepares the embryonic lung for inflation at birth. It is well-documented that the late gestation embryo performs episodic breathing-like movements called as fetal breathing movements (FBMs), but the physiological importance of these events is not clear. Here we aimed to study the physiological role of FBMs in preparation for air inflation at birth. *Clp1*^*K/K*^ late gestation embryos develop a progressive loss of spinal motor neurons associated with axonal degeneration and denervation of neuromuscular junctions serving as an ideal genetic model to test the possible role of FBMs. We demonstrated that *Clp1*^*K/K*^ newborns show impaired motor function resulting in fatal respiratory failure after birth. Next, we showed that the alveolar septa are thicker, and the alveolar area is reduced in *Clp1*^*K/K*^ late gestation embryos, while the expression of molecular markers of lung development are not affected. Importantly, pulmonary lymphatic vessels are dilated and the prenatal pulmonary lymphatic function is reduced in *Clp1*^*K/K*^ late gestation embryos. Our results have revealed that *Clp1*^*K/K*^ mice show impaired motor functions including FBMs, and late gestation *Clp1*^*K/K*^ embryos display reduced prenatal lymphatic function and impaired lung expansion represented as thickened alveolar septa and reduced alveolar area in preparation of the developing lung for inflation at birth. These findings suggest a possible mechanism that FBMs, similarly to breathing movements after birth, stimulate prenatal lymphatic function in pulmonary collecting lymphatics lacking smooth muscle coverage to prepare the developing lung for inflation and gas exchange at birth. Moreover, these results raise the possibility that stimulating FBMs during late gestation might be an effective way to reduce the risk of the development of neonatal respiratory failure.

## Introduction

Embryonic lungs develop in a fluid compartment *in utero* and must be inflated immediately after birth to establish respiration. A complex developmental program prepares the fluid-filled embryonic lung for the postnatal life (Morrisey and Hogan, 2010; Nikolic et al., 2018; Whitsett et al., 2019). After the pulmonary developmental program is completed the first breaths require much greater forces to inflate the lung and establish respiration than the subsequent breathing movements (Milner and Sauders, 1977; Hall, 2016). Pulmonary surfactant secreted by type II pneumocytes is a well-known regulator of pulmonary compliance and lung inflation at birth (Morgan, 1971). In connection, the current therapeutic approaches mainly focus on inducing and stimulating surfactant production and the possible ways of surfactant substitution in preterm infants and newborns with respiratory distress syndrome (Couser et al., 1990; Nouraeyan et al., 2014; Polin and Carlo, 2014).

In addition to surfactant, our recent studies have revealed the importance of prenatal lymphatic function as a regulator of lung inflation at birth by inducing the expansion and increasing the compliance of the developing lung in preparation for the first breath (Jakus et al., 2014). It was shown before that LECs are present in the developing lung at E12.5, which form lumen by E14.5 in mouse embryos (Kulkarni et al., 2011), but the function of these lymphatic structures was unclear. Our studies indicated that parallel mechanisms, which include prenatal lymphatic function are involved in the preparation of the prenatal lung for inflation at birth (Jakus et al., 2014; Aspelund et al., 2016). Recent studies demonstrated that the structure of the pulmonary collecting lymphatic vessels is different compared to other lymphatic collectors in different organs, which are covered by smooth muscle cells providing the pumping function and maintaining the forward flow of the lymph fluid with the help of the lymphatic valves (Maby-El Hajjami and Petrova, 2008). In contrast to the other organs the collecting lymphatics in the lung do not have smooth muscle coverage (Outtz Reed et al., 2019). Therefore, it is essential to have an independent mechanism in the lung which provides the pumping function and maintains the lymph flow. Breathing is always present in the adult lung to stimulate the lymph flow of pulmonary lymphatic vessels (Outtz Reed et al., 2019), but it is an open question how lymph flow is induced and maintained in the developing lung in the lack of smooth muscle coverage of collecting lymphatics.

Early studies suggested that fluid secretion and mechanical forces influence the expansion and developmental process of the embryonic lung (Perlman et al., 1976; Wigglesworth and Desai, 1982; Moessinger et al., 1990; Harding and Hooper, 1996; Kitterman, 1996). It has been shown that prolonged leakage of the amniotic fluid results in a collapsed lung phenotype, which was referred as lung hypoplasia (Perlman et al., 1976). In connection to this, a more recent study indicated that mechanical forces induced by amniotic fluid inhalation influence the differentiation of type II and type I pneumocytes (Li et al., 2018).

It is well known that the late gestation embryo performs periodic breathing-like movements during late gestation which are called as FBMs. In connection to their possible function, altered lung development was detected in human fetuses with skeletal muscle defects, which changes of lung morphology were referred as lung hypoplasia (Sandler et al., 1994). However, these paralyzed embryos appeared to be much smaller than the embryos with normal skeletal muscle function, which finding indicates that the defect of skeletal muscle innervation starting from the early life period significantly influences the size and growth rate of the embryo. Therefore, based on these prior studies the possible physiological role of periodic FBMs during late gestation remains unclear.

To define the role of FBMs on prenatal lung development, surgical approaches including phrenic nerve section and spinal cord transection were also applied in lamb embryos (Fewell et al., 1981; Liggins et al., 1981). These heroic studies involving the surgical manipulation of the embryos in the womb indicated that transection of the spinal cord above the phrenic nucleus or the section of phrenic nerve results in reduced lung expansion with reduced wet lung weights (Fewell et al., 1981; Liggins et al., 1981). Based on the results the authors concluded that the lack of FBMs leads to lung hypoplasia, which became a widely accepted paradigm. However, if we analyze these studies in detail we will find that the dry weights of the lungs were not reduced after the surgical procedures, questioning the main concept about lung hypoplasia (Fewell et al., 1981). In addition, reports aiming to repeat the phrenic nerve section studies in more controlled experiments in lambs found that the complicated surgical procedure in sham operated animals has more pronounced effect on the morphology of the developing lung than the phrenic nerve section itself (Bamford et al., 1992). Therefore, the authors concluded that phrenic nerve section does not cause the hypoplasia of the developing lung (Bamford et al., 1992). Important to note that not only large animals and humans but mouse embryos also perform FBMs during late gestation (Niblock et al., 2020). As another approach, a mouse model was reported in which myogenin deficiency leads to the disruption of normal skeletal muscle structure (Tseng et al., 2000). The authors concluded the presence of pulmonary hypoplasia in this model, but it is important to consider that the skeletal muscles are affected from the early embryonic period, therefore not only the FBMs but the overall growth rate of the embryo is also altered in the model (Tseng et al., 2000).

Based on the above studies it is suggested that mechanical forces generated by FBMs influence the developmental process of the prenatal lung. However, all the experimental systems used in these prior studies answer different questions or have great limitations. Leakage or drainage of the amniotic fluid may affect not only FBMs but also the volume and function of the organs, embryos paralyzed from the early embryonic period show severe defects of the skeleton which is represented in their smaller size and reduced growth, and surgical approaches destroying the spinal cord or phrenic nerve are heroic and may influence a number of physiological processes in addition to FBMs.

Recently Hanada et al. (2013) studied the phenotype of *Clp1*^*K/K*^ embryos carrying a kinase-dead CLP1 RNA kinase, these embryos develop a progressive loss of spinal motor neurons associated with axonal degeneration and denervation of neuromuscular junctions at E16.5 onward including the respiratory skeletal muscles (diaphragm etc.). The denervation causes impaired motor function, weakness of the skeletal muscles, paralysis, and respiratory failure at birth (Hanada et al., 2013). It has also been demonstrated that CLP1 has an important function in the motor neurons, where the loss of CLP1 activity induces the accumulation of small RNA fragments because of the abnormal processing of tyrosine pre-transfer RNA. The elevated levels of the tRNA fragments lead to oxidative-stress-mediated p53 activation and p53-dependent elimination of motor neurons (Hanada et al., 2013).

Herein, we aimed to use the *Clp1*^*K/K*^ model as a genetic model with skeletal muscle denervation including the diaphragm at E16.5 onward (Hanada et al., 2013) to test the possible role of FBMs in preparation for air inflation at birth in experimental mice. Our studies indicate that *Clp1*^*K/K*^ embryos displaying impaired motor functions and FBMs show reduced prenatal lung expansion without significantly affecting the molecular and cellular lung development during the embryonic period. Importantly, our results suggest a possible mechanism that one of the main functions of FBMs is to stimulate prenatal pulmonary lymphatic function in pulmonary lymphatic vessels lacking smooth muscle coverage and increase prenatal lung expansion in preparation for lung inflation at birth.

## Materials and Methods

### Animals

Mice carrying the kinase-dead Clp1 allele (*Clp1*^*K*^) (Hanada et al., 2013) were maintained on a c57Bl/6 and a c57Bl/6-NMRI mixed genetic backgrounds. The embryos and newborns of heterozygous matings were genotyped by allele-specific PCR using 5′-TTG GTT CAG GTA TTA AGT CGT TGG-3′ forward and 5′-GAA TTG CAT AGT CTT TCC TCC ATC-3′ reverse primers. *Flt4*^*YFP*^ mice (Calvo et al., 2011) were crossed to *Clp1*^*K/+*^ animals and maintained in heterozygous form on a c57Bl/6 background. The offspring were genotyped by allele-specific PCR primer sets including 5′-GGA TCA CTC TCG GCA TGG AC-3′ forward and 5′-GGG CGT CCT CAT ACC TAG GT-3′ reverse primers.

Experimental animals were housed in either specific pathogen free or conventional animal facilities. All animal experiments were approved by the Animal Experimentation Review Board of the Semmelweis University and the Government Office for Pest County (Hungary).

### Timed Matings and Handling of Late Gestation Embryos and Newborns

*Clp1*^*K/+*^ heterozygous animals were used to set up overnight timed matings. To examine prenatal lung morphology and development before air exposure and extra-uterine respiratory changes, the embryos were sacrificed *in utero* by immersing the gravid uterus into ice-cold PBS for 40 min before harvesting the embryos from the uterus under fluid as described before (Jakus et al., 2014).

Embryos were collected at E14.5, E15.5, E16.5, E17.5, E18.5, and E19.5. Cesarean sections were performed at E19.5 followed by the rapid removal of the embryos from the uterus and their manual stimulation. Naturally born and cesarean section newborns were monitored for 2 h after birth and scored after 20–30 min. A skeletal muscle activity score (0–5) system was used in which the general skeletal muscle activity of the newborn was assessed including the movements [no movements or only after stimulation, spontaneous limited movements, poor motor activity, good motor activity, and excellent motor activity (0–4)] and respiration [no breathing or gasping, normal breathing (0–1)]. A viability score system (0–7) was also used in which the newborns were scored based on their activity and movements [no movements or only after stimulation, spontaneous limited movements, poor motor activity, good motor activity, and excellent motor activity (0–4)], appearance [cyanotic or normal color (0–1)], breathing [no breathing or gasping, normal breathing (0–1)], and breathing activity 60 min post birth (0–1). Thereafter, tail samples were collected for genotyping, and tissues were harvested from the embryos and newborns. The whole chest, isolated lungs, gut, and skin were used for histology, weight, and DNA content measurements.

### Histological Processes and Immunohistochemistry

Embryonic and newborn tissues were fixed in 4% paraformaldehyde (Sigma-Aldrich) overnight on 4°C, dehydrated in 50, 70, 95, and 100% ethanol, then embedded in paraffin using a Leica EG1150H embedding station. Seven-micrometer-thick sections were generated using a HM340E Thermo Scientific microtome, and processed for hematoxylin–eosin (HE) (Leica), periodic acid-Schiff (PAS) (Sigma-Aldrich), trichrome (Sigma-Aldrich), and immunohistochemistry staining. The following primary antibodies were used for immunostaining: anti-LYVE1 (R&D Systems, AF2125), anti-PROX1 (Angiobio, 11-002P), anti-VEGRF3 (R&D, AF743) anti-CC10 (Santa Cruz Biotechnology, Inc., sc-9772), anti-proSPC (Merck, AB3786), anti-α-SMA (Abcam, ab124964), anti-Desmin (Dako, M0760), anti-PDPN (R&D Systems, AF3244), anti-PDGFRα (Cell Signaling Technology, 3164), anti-PDGFRβ (Cell Signaling Technology, 3169), anti-Vimentin (Cell Signaling Technology, 5741), anti-PECAM1 (R&D Systems, MAB3628), and anti-NG2 (EMD Millipore, AB5320). As secondary antibodies Alexa Fluor 488 and 568 conjugated anti-goat or anti-rabbit antibodies (Life Technologies) were used. As a nucleus staining DAPI containing mounting medium (Vector Laboratories) was used. Microscopic images were taken by a Nikon ECLIPSE Ni-U microscope connected to a Nikon DS-Ri2 camera. Alveolar area (averaging 8–10 fields of view per embryo) and septal thickness (averaging 80–100 measurements per embryo), lymphatic vessel area (average of all visible pulmonary lymphatic vessels per embryo per section and normalized for the mean area of the littermate controls) measurements were performed in NIS-Elements Imaging Software (Nikon) using a 40× dry objective (40× images). Different structures and cell types of immunofluorescent images were quantified using Fiji software (Schindelin et al., 2012). PDPN positive cells, Desmin positive cells, PDGFRα positive cells, NG2 positive cells, and Vimentin positive cells were counted on an area of 100 μm ^∗^ 100 μm. CC10 positive cells, SPC positive cells, LYVE1 and PROX1 positive structures, PDGFRβ positive structures, and VEGFR3 positive structures were counted on the whole field of view of 40× images. PECAM positive structures and α-SMA positive vascular structures were counted on the whole field of view of 20× images. Alveolar area was measured using “Freehand selections” tool and was extracted from the area of the examined area thus the area of the interstitial tissue calculated. All cell counts (CC10, SPC, PDPN, PDGFRα, NG2, Desmin, and Vimentin) were normalized to this calculated interstitial area. Vascular structure counts (PDGFRβ, α-SMA, NG2, PECAM1, VEGFR3, PROX1, and LYVE1) are shown as mean and SEM.

### DNA Content Measurements

For total DNA content measurements, DNA was isolated from whole lungs of *Clp1*^*K/K*^ and littermate control E18.5 embryos using DNeasy blood and tissue isolation kit (Qiagen). DNA concentration of the samples was determined by NanoDrop OneC Microvolume UV–Vis Spectrophotometer (Thermo Scientific).

### Monitoring Pulmonary Lymphatic Function *in vivo*

To monitor lymphatic function pregnant *Clp1*^*K/+*^ females time-mated with *Clp1*^*K/+*^ males were anesthetized. 0.5 μl of 70 kDa RhD (Life Technologies) at 10 mg/ml concentration was injected through the uterus and chest wall into the lung of E18.5 *Clp1*^*K/K*^ and littermate control embryos on *Flt4*^*YFP*^ lymphatic reporter background as described before (Jakus et al., 2014; Bálint et al., 2020). Selective uptake and transport of large molecular weight RhD in fluorescent reporter positive lymphatic vessels were monitored 60 min after the injection by a fluorescent Nikon SMZ25 stereomicroscope equipped with Nikon DS-Ri2 camera. To quantify the transport of fluorescently labeled macromolecules, the intensity of RhD signal was measured in NIS-Elements Imaging Software (Nikon) in reporter positive lymphatic vessels (the background intensity was subtracted). After imaging, the tissue samples were collected for genotyping.

### Monitoring FBMs in *Clp1*^*K/K*^ and Littermate Control Embryos *in utero*

Pregnant *Clp1*^*K/+*^ mice time-mated with *Clp1*^*K/+*^ males were anesthetized by isoflurane. Medial laparotomy was performed in a supine position and the uterus was carefully externalized. Embryos of pregnant mice were scanned by using a micro-ultrasound imaging unit (Visualsonics, Vevo 3100 imaging system) equipped with an ultrahigh frequency MX400 transducer (30 MHz, 55 frames per second) at E18.5. Sudden displacement of the diaphragm was considered as a FBM when it was followed by expansion of the ribcage. Two minutes long videos were recorded and FBMs were counted by a blinded, trained observer. Tissue samples from embryos were collected for genotyping after scanning.

### Presentation of Data and Statistical Analysis

Experiments were performed the indicated number of times. Macroscopic pictures and microscopic images are representative of three or more independent experiments. For all experiments, investigators were blinded for the origin of embryos and newborns until the end of the analysis. Image processing and analysis were performed using NIS-Elements Imaging (Nikon), Fiji Software (NIH), and Adobe Photoshop. Results are shown as mean and SEM. Statistical analyses were performed using GraphPad Prism 7.0 and Microsoft Office Excel software programs. Specific statistical tests are presented in the figure legend for each experiment. *P*-values <0.05 were considered statistically significant.

## Results

### *Clp1*^*K/K*^ Embryos and Newborns Exhibit Impaired Skeletal Muscle Function Including FBMs, Cyanosis, and Die Shortly After Birth

**FIGURE 1:**
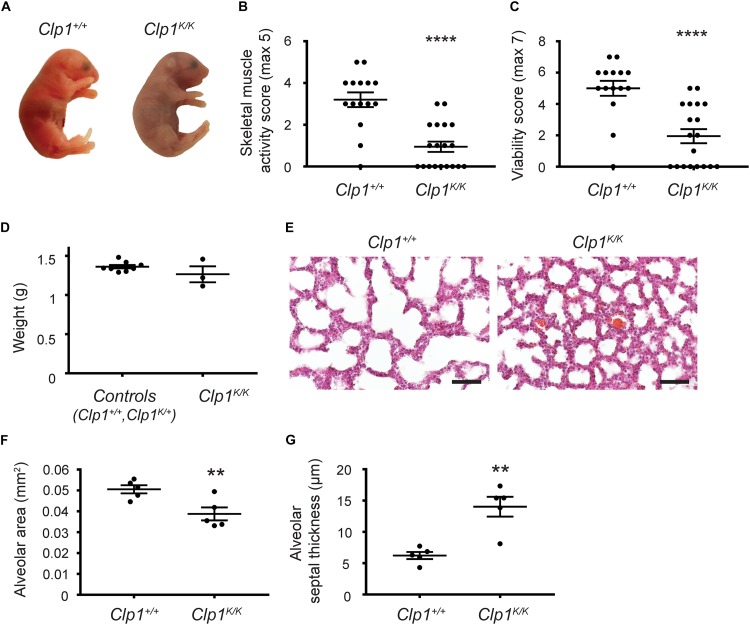
*Clp1*^*K/K*^ newborns show impaired skeletal muscle activity, develop respiratory failure, and die after birth. **(A)** Appearance of newborn *Clp1*^+/+^ and *Clp1*^*K/K*^ littermates on a c57Bl/6 genetic background. Representative images are shown of 15–19 embryos from six litters. **(B)** Overall skeletal muscle activity score of newborn *Clp1*^+/+^ and *Clp1*^*K/K*^ littermates on a c57Bl/6 genetic background. Quantitative data are shown as mean and SEM of 15–19 embryos from six litters [^****^P < 0.0001 (two-tailed *t*-test)]. **(C)** Viability score of newborn *Clp1*^+/+^ and *Clp1*^*K/K*^ littermates on a c57Bl/6 genetic background. Quantitative data are shown as mean and SEM of 15–19 embryos from six litters [^****^P < 0.0001 (two-tailed *t*-test)]. **(D)** Total weight of control (*Clp1*^+/+^ or *Clp*^*K/+*^) and *Clp1*^*K/K*^ newborns. Quantitative data are shown as mean and SEM of three to eight embryos from one litter [*P* = 0.1868 (two-tailed *t*-test)]. **(E)** Representative images of lung morphology shown by HE staining of newborn *Clp1*^+/+^ and *Clp1*^*K/K*^ littermates on a c57Bl/6 background 60 min after birth. Representative images are shown of five newborns of each group. Bars, 50 μm. **(F,G)** Quantitative data for alveolar area **(F)** and alveolar septal thickness **(G)** are represented in newborn lungs of *Clp1*^+/+^ and *Clp1*^*K/K*^ littermates on a c57Bl/6 genetic background [mean and SEM, *n* = 5 per group, ^∗∗^*P* = 0.0090 for alveolar area and ^∗∗^*P* = 0.0030 for septal thickness (two-tailed *t*-test)].

*Clp1*^*K/K*^ mice carrying a kinase dead CLP1 RNA kinase were reported to develop a progressive loss of motoneurons on the c57Bl/6 genetic background (Hanada et al., 2013). First, we tested the phenotype of *Clp1*^*K/K*^ newborns. As expected, the *Clp1*^*K/K*^ newborns showed cyanosis, signs of acute respiratory failure, and died shortly after natural birth or cesarean section (most of them within 30 min) (Figure 1A). After birth, the skeletal muscle function in *Clp1*^*K/K*^ newborns was greatly impaired compared to the control littermates as it is represented in Supplementary Movie S1. In accordance, the skeletal muscle activity score and the viability score of *Clp1*^*K/K*^ newborns were also greatly reduced compared to the littermate controls (^****^P = 6.94 ^∗^ 10^–6^ for skeletal muscle activity score and ^****^P = 5.92 ^∗^ 10^–5^ for viability) (Figures 1B,C). Of note, the visible size (Figure 1A) and measured weight (*P* = 0.1868) (Figure 1D) of *Clp1*^*K/K*^ newborns were not reduced compared to the littermate control newborns. HE staining of the newborn lungs revealed unaltered structure with reduced alveolar area and thickened alveolar wall in the lung of *Clp1*^*K/K*^ newborns (^∗∗^*P* = 0.0090 for alveolar area and ^∗∗^*P* = 0.0030 for septal thickness) (Figures 1E–G). FBMs have been described in mouse embryos during late gestation, but effectively monitoring them in mouse genetic models has great limitations (Niblock et al., 2020; and discussed below). Despite these limitations we observed FBMs in *Clp1*^+/+^ and *Clp1*^*K/+*^ control embryos at E18.5 [2.67 ± 1.12 FBMs in 2 min (mean and SEM); five out of six control embryos performed FBMs], and no FBM was detectable in 1 *Clp1*^*K/K*^ embryo with normal heart activity in one litter (Supplementary Movies S2, S3). Importantly, it has been reported that breathing-like episodes correlate well with the breathing activity and movements of the newborn after birth (Niblock et al., 2020). Collectively, our results confirmed that *Clp1*^*K/K*^ genetic model with impaired skeletal muscle function involving the respiratory muscles is an excellent tool to study the possible role of mechanical forces and FBMs during the late gestation period.

### Late Gestation *Clp1*^*K/K*^ Embryos Do Not Exhibit Altered Expression of Molecular Markers of Lung Development

**FIGURE 2:**
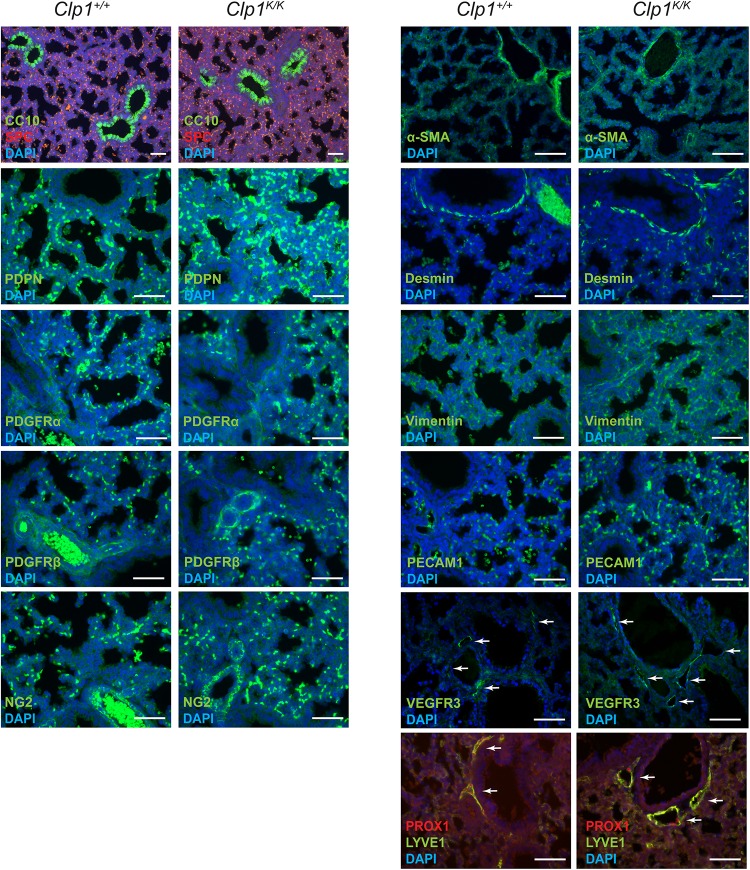
Normal molecular and cellular lung development in late gestation *Clp1*^*K/K*^ embryos. Immunostaining for CC10, SPC, PDPN, PDGFRα, PDGFRβ, NG2, α-SMA, Desmin, Vimentin, PECAM1, VEGFR3, PROX1, LYVE1, and DAPI nuclear staining are shown in late gestation *Clp1*^+/+^ and *Clp1*^*K/K*^ embryonic lungs at E18.5. Representative images are shown of three to five independent experiments per group. Bars, 50 μm.

Next, we characterized the molecular lung development in *Clp1*^*K/K*^ embryos on the c57Bl/6 genetic background. Late lung developmental markers for lung Club (Clara) cells (CC10), alveolar type II cells (SPC), type I cells (PDPN), mesenchyme (PDGFRα, Vimentin, Desmin), vascular smooth muscle cells and pericytes (PDGFRβ, NG2, and α-SMA), lung endothelial cells (PECAM1), and pulmonary LECs (VEGFR3, PROX1, and LYVE1) showed normal expression levels with no major difference in the number of cells or vascular structures in *Clp1*^*K/K*^ embryos compared to the *Clp1*^+/+^ littermates at E18.5 shown by representative images and quantification (Figure 2 and Supplementary Table S1). Collectively, analyzing the expression of cellular and molecular lung developmental markers indicated unaltered pulmonary development in *Clp1*^*K/K*^ embryos. The only observed alteration in late gestation *Clp1*^*K/K*^ embryos was the marked dilation of pulmonary lymphatic vessels in the embryonic lungs compared to the control lungs (note the arrows in the figure panels) shown by LYVE1, PROX1, and VEGFR3 lymphatic markers (Figure 2).

### Late Gestation Lung of *Clp1*^*K/K*^ Embryos With Impaired Skeletal Muscle Function Shows Thickened Alveolar Septum and Reduced Alveolar Area Before Air Inflation

**FIGURE 3:**
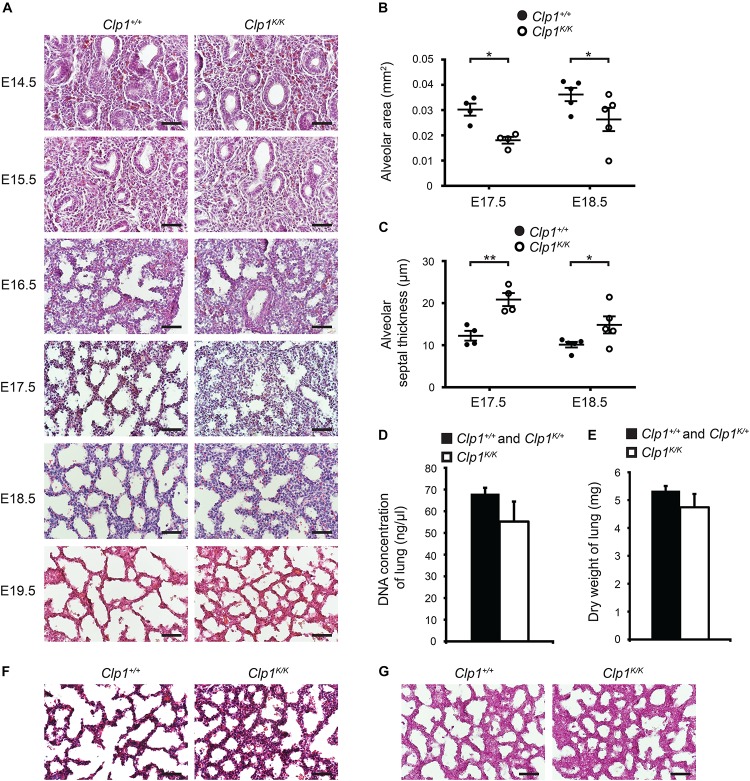
Thicker alveolar septum and reduced alveolar area in *Clp1*^*K/K*^ embryos before air inflation of the lung. **(A)** Lung morphology shown by HE lung histology of *Clp1*^+/+^ and *Clp1*^*K/K*^ embryos before air inflation *in utero* at E14.5, E15.5, 16.5, E17.5, E18.5, and E19.5 on a c57Bl/6 genetic background. Representative images are shown of two to four embryos per group from one to two litters. Bars, 50 μm. **(B,C)** Quantitative data for alveolar area **(B)** and alveolar septal thickness **(C)** of *Clp1*^+/+^ and *Clp1*^*K/K*^ embryonic lungs (E17.5 and E18.5) on a c57Bl/6 genetic background. Quantitative data are represented as mean and SEM from four to five embryos in each group [^∗^*P* = 0.0165 at E17.5 and ^∗^*P* = 0.0103 at E18.5 for alveolar area and ^∗∗^*P* = 0.0018 at E17.5 and ^∗^*P* = 0.0348 at E18.5 for septal thickness (two-tailed *t*-test)]. **(D)** Total DNA content of the lung shown as DNA concentration in late gestation control (*Clp1*^+/+^ and *Clp1*^*K/+*^) and *Clp1*^*K/K*^ embryos at E18.5. Quantitative data are shown as mean and SEM from five to six embryos in each group [*P* = 0.1753 (two-tailed *t*-test)]. **(E)** Dry weights of lung of late gestation control (*Clp1*^+/+^ and *Clp1*^*K/+*^) and *Clp1*^*K/K*^ embryos at E18.5. Quantitative data are shown as mean and SEM from 5 to 17 embryos in each group [*P* = 0.1452 (two-tailed *t*-test)]. **(F,G)** Trichrome staining **(F)** and periodic acid-Schiff (PAS) staining **(G)** for levels of the surfactant precursor glycogen in the lungs of *Clp1*^+/+^ and *Clp1*^*K/K*^ embryos at E18.5. Representative images are shown of three embryos examined in each group. Bars, 50 μm.

To study the possible impact of FBMs on the structure of the developing lung, lung morphology in *Clp1*^*K/K*^ embryos revealed no detectable changes before E16.5 compared to the controls (Figure 3A). We observed increased septal wall thickness and reduced alveolar space area at E17.5 compared to the controls, and these changes were also present at E18.5 *in utero* before air inflation of the lung in *Clp1*^*K/K*^ embryos (^∗^*P* = 0.0165 for alveolar area and ^∗∗^*P* = 0.0018 for septal thickness at E17.5 and ^∗^*P* = 0.0103 for alveolar area and ^∗^*P* = 0.0348 for septal thickness at E18.5) (Figures 3A–C). Of note, the DNA content and dry weights of the *Clp1*^*K/K*^ embryonic lungs at E18.5 were not reduced compared to control littermates, which results are consistent with our molecular findings supporting normal growth and maturation of lung cell types (*P* = 0.1753 for DNA content and *P* = 0.1452 for dry weight) (Figures 3D,E and Supplementary Table S1). Trichrome staining and PAS staining also revealed normal lung structure in *Clp1*^*K/K*^ embryos but alveolar interstitial thickness was increased and the alveolar area was reduced compared to the control at E18.5 (Figures 3F,G). Glycogen as a surfactant precursor accumulates in the developing lung if surfactant production is blocked (Bourbon et al., 1982). Importantly, PAS staining indicated normal levels of glycogen in the alveolar cells of *Clp1*^*K/K*^ embryonic lungs at E18.5 (Figure 3G).

Collectively, the studies of the *Clp1*^*K/K*^ genetic model suggest the importance of FBMs in the regulation of lung morphology and expansion during late gestation, but not influencing the cellularity and growth of the embryonic lung. Of note, the observed morphological changes in *Clp1*^*K/K*^ embryos displaying impaired FBMs are very similar to the phenotype of lymphatic-deficient embryos (Jakus et al., 2014).

### The Pulmonary Lymphatic Vessels Are Markedly Dilated in Late Gestation *Clp1*^*K/K*^ Embryos With Impaired Skeletal Muscle Function Before Air Inflation

**FIGURE 4:**
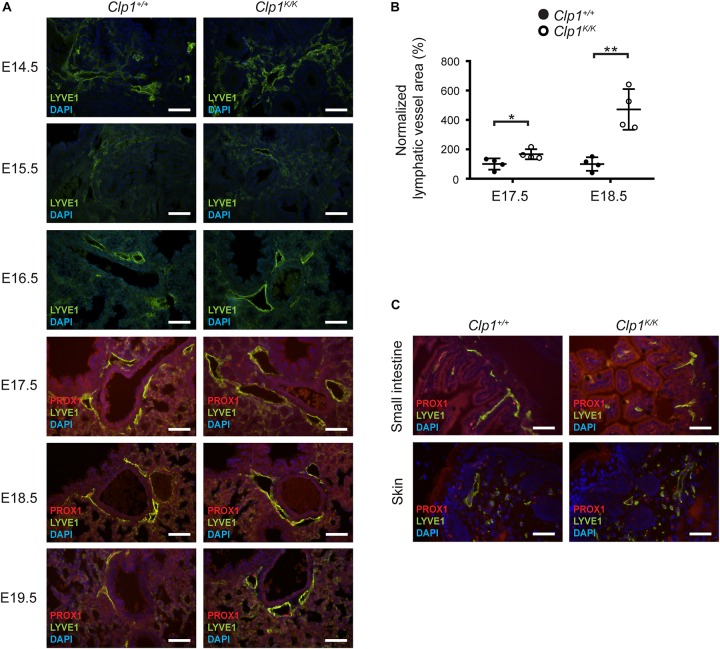
Dilated pulmonary lymphatic vessels in late gestation *Clp1*^*K/K*^ embryos. **(A)** Morphology of lymphatic vessels shown by immunostaining for LYVE1, PROX1 lymphatic markers, and DAPI nuclear staining at different stages of embryonic life (E14.5, E15.5, 16.5, E17.5, E18.5, and E19.5) of *Clp1*^+/+^ and *Clp1*^*K/K*^ embryonic lungs on a c57Bl/6 genetic background. Representative images are shown of two to four embryos per each group. Bars, 50 μm. **(B)** Normalized lymphatic vessel area in *Clp1*^+/+^ and *Clp1*^*K/K*^ embryos at E17.5 and E18.5 before air inflation on a c57Bl/6 genetic background. Quantitative data are shown as mean and SEM of four embryos in each group from three litters [^∗^*P* = 0.0443 at E17.5; ^∗∗^*P* = 0.0073 at E18.5 (two-tailed *t*-test)]. **(C)** Lymphatic morphology shown by immunostaining for LYVE1, PROX1, and nuclear DAPI staining in the skin and small intestine of *Clp1*^+/+^ and *Clp1*^*K/K*^ embryos at E18.5 on a c57Bl/6 genetic background. Representative images are shown of four embryos from three litters. Bars, 50 μm.

Developing pulmonary lymphatic structures can be detected at E14.5 in the embryonic lung (Figure 4A). Importantly, the lymphatic vessels showed marked dilation at E17.5 in *Clp1*^*K/K*^ embryos, and the dilation was more apparent at E18.5 and E19.5 compared to the littermate controls (^∗^*P* = 0.0443 at E17.5; ^∗∗^*P* = 0.0073 at E18.5) (Figures 4A,B). In contrast, the structure of the lymphatic vessels showed no difference compared to the littermate controls in other organs including the small intestine and the skin (Figure 4C). These results indicate that the lymphatics are present and show unaltered development in *Clp1*^*K/K*^ embryos, but the lymphatic structures appear to be markedly dilated in *Clp1*^*K/K*^ embryos, which may indicate the impairment of pulmonary lymphatic function.

### The Presence of Lymphatic Function Impairment in Late Gestation Lungs of *Clp1*^*K/K*^ Embryos

**FIGURE 5:**
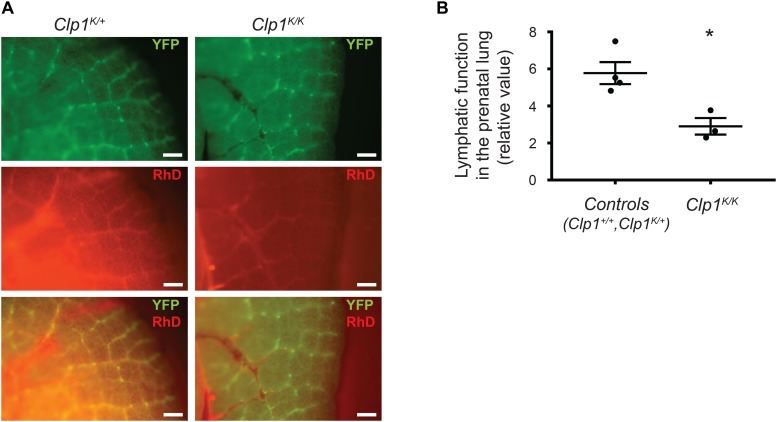
Impaired pulmonary lymphatic function in late gestation *Clp1*^*K/K*^ embryos. **(A)** Monitoring the uptake and transport of fluorescently labeled macromolecules by pulmonary lymphatic vessels in control (*Clp1*^*K/+*^) and *Clp1*^*K/K*^ E18.5 embryonic lungs carrying *Flt4*^*YFP*^ lymphatic reporter allele on a c57Bl/6 genetic background injected with 70 kDa RhD into the lung through the chest wall and uterus. Representative images are shown of three to four embryos per group from five litters. Bars, 250 μm. **(B)** Quantitative data for the uptake of fluorescently labeled macromolecules (RhD) into YFP positive lymphatic vessels (intensity of RhD signal in fluorescently labeled pulmonary lymphatic vessels, the background intensity was subtracted) are shown as mean and SEM at E18.5 in control (*Clp1*^+/+^, *Clp1*^*K/+*^) and *Clp1*^*K/K*^ embryos from three to four embryos per each group from five litters [^∗^*P* = 0.0151 (two-tailed *t*-test)].

To monitor the pulmonary lymphatic function in late gestation embryos, 70 kDa RhD was injected into the embryonic lung of *Clp1*^*K/K*^ and littermate control embryos on the *Flt4*^*YFP*^ lymphatic reporter genetic background at E18.5 through the uterine wall and chest wall. Selective uptake and transport of the fluorescently labeled macromolecule in reporter positive pulmonary lymphatic vessels was detectable 60 min post injection in *Clp1^*K/*+^* control embryos. Importantly, the uptake and transport of the labeled macromolecule in the YFP positive lymphatic vessel indicating the lymphatic function was severely reduced in *Clp1*^*K/K*^ embryos at E18.5 compared to littermate controls (^∗^*P* = 0.0151) (Figures 5A,B).

### Postnatal Phenotype of *Clp1*^*K/K*^ Newborns on a Mixed Genetic Background

**FIGURE 6:**
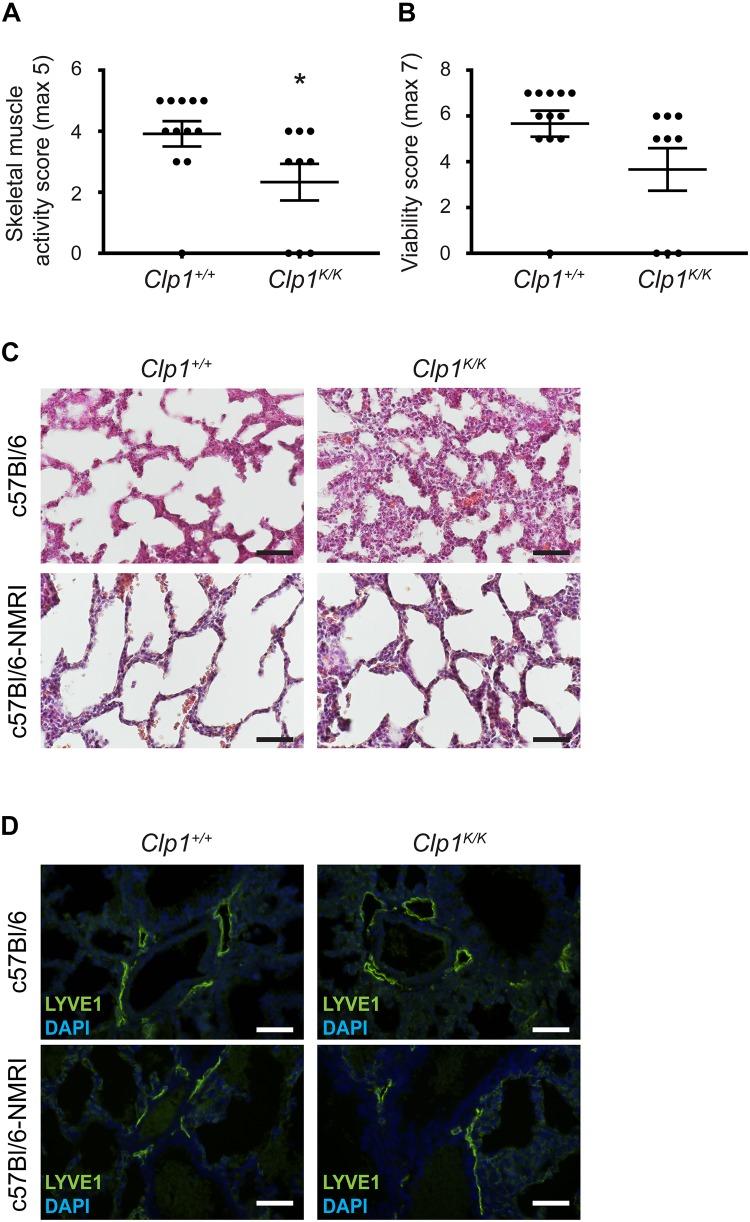
Postnatal phenotype of *Clp1*^*K/K*^ newborns on a c57Bl/6-NMRI mixed genetic background. **(A)** Overall skeletal muscle activity score of newborn *Clp1*^+/+^ and *Clp1*^*K/K*^ littermates on a c57Bl/6-NMRI mixed genetic background. Quantitative data are represented as mean and SEM from 9 to 12 newborns in each group [^∗^*P* = 0.0374 (two-tailed *t*-test)]. **(B)** Viability score of newborn *Clp1*^+/+^ and *Clp1*^*K/K*^ littermates on a c57Bl/6-NMRI mixed genetic background. Quantitative data are represented as mean and SEM from 9 to 12 newborns in each group [*P* = 0.0684 (two-tailed *t*-test)]. **(C)** Lung morphology of newborn *Clp1*^+/+^ and *Clp1*^*K/K*^ littermates on c57Bl/6 and c57Bl/6-NMRI mixed genetic backgrounds shown by HE histology. Representative images are shown from seven newborns from three litters. Bars, 50 μm. **(D)** Visualization of pulmonary lymphatics in *Clp1*^+/+^ and *Clp1*^*K/K*^ newborns on c57Bl/6 and c57Bl/6-NMRI mixed genetic backgrounds. Representative images are shown for LYVE1 immunostaining and DAPI from five newborns per group in two litters. Bars, 50 μm.

It is thought that the phenotype of transgenic animals tends to be less severe on a mixed genetic background than on a pure inbred genetic background. In connection, it is documented that lymphatic phenotypes are less severe on the NMRI genetic background, serving as a permissive genetic background (Karkkainen et al., 2001). Therefore, to study the possible correlation of the skeletal muscle activity in late gestation embryos with the prenatal lung phenotype and lymphatic morphology we crossed the *Clp1*^*K/K*^ genetic model to a c57Bl/6-NMRI mixed genetic background. First, we characterized the motility and viability of *Clp1*^*K/K*^ newborns on this genetic background (Figures 6A,B). The clinical score representing the overall skeletal muscle activity score was reduced in *Clp1*^*K/K*^ newborns on the c57Bl/6-NMRI mixed background compared to the control newborn mice [59.57 ± 15.34% normalized to the controls (mean and SEM)] (^∗^*P* = 0.0374) (Figure 6A). The clinical score representing viability of the *Clp1*^*K/K*^ newborns was also reduced on the c57Bl/6-NMRI mixed background compared to the controls but this difference was not significant [64.7 ± 16.38% normalized to the controls (mean and SEM)] (*P* = 0.0684) (Figure 6B). Note that the overall skeletal muscle activity score of *Clp1*^*K/K*^ newborns on the pure c57Bl/6 genetic background is 29.6 ± 7.73% (mean and SEM) normalized to the controls (^****^P = 6.94 ^∗^ 10^–6^), and the clinical score representing viability of the *Clp1*^*K/K*^ newborns is 38.95 ± 8.98% (mean and SEM) normalized to the controls (^****^P = 5.92 ^∗^ 10^–5^) (Figures 1B,C). These results indicate that there is a trend suggesting that the newborn muscle function and viability is less impaired in *Clp1*^*K/K*^ newborns on c57Bl/6-NMRI mixed genetic background than on the pure c57Bl/6 background. Moreover, the lung morphological changes were also less severe on the c57Bl/6-NMRI mixed genetic background than on the c57Bl/6 background in *Clp1*^*K/K*^ newborns (Figure 6C). Importantly, 3 *Clp1*^*K*/*K*^ mice out of 134 pups in 15 litters survived to P21 on c57Bl/6-NMRI mixed background, while no *Clp1*^*K*/*K*^ mouse was found to survive to P21out of 86 mice in 19 litters on the pure c57Bl/6 genetic background. These comparisons indicate that the skeletal muscle function is more intact in *Clp1*^*K/K*^ newborns on the c57Bl/6-NMRI mixed background than it is on the pure c57Bl/6 genetic background. Importantly, the lymphatic vessels are less dilated in *Clp1*^*K/K*^ newborns on the c57Bl/6-NMRI mixed background if we compare them to the pulmonary lymphatic vessels in *Clp1*^*K/K*^ newborns on the c57Bl/6 genetic background (Figure 6D).

### Prenatal Phenotype of *Clp1*^*K/K*^ Embryos on a Mixed Genetic Background With Less Impaired Skeletal Muscle Function

**FIGURE 7:**
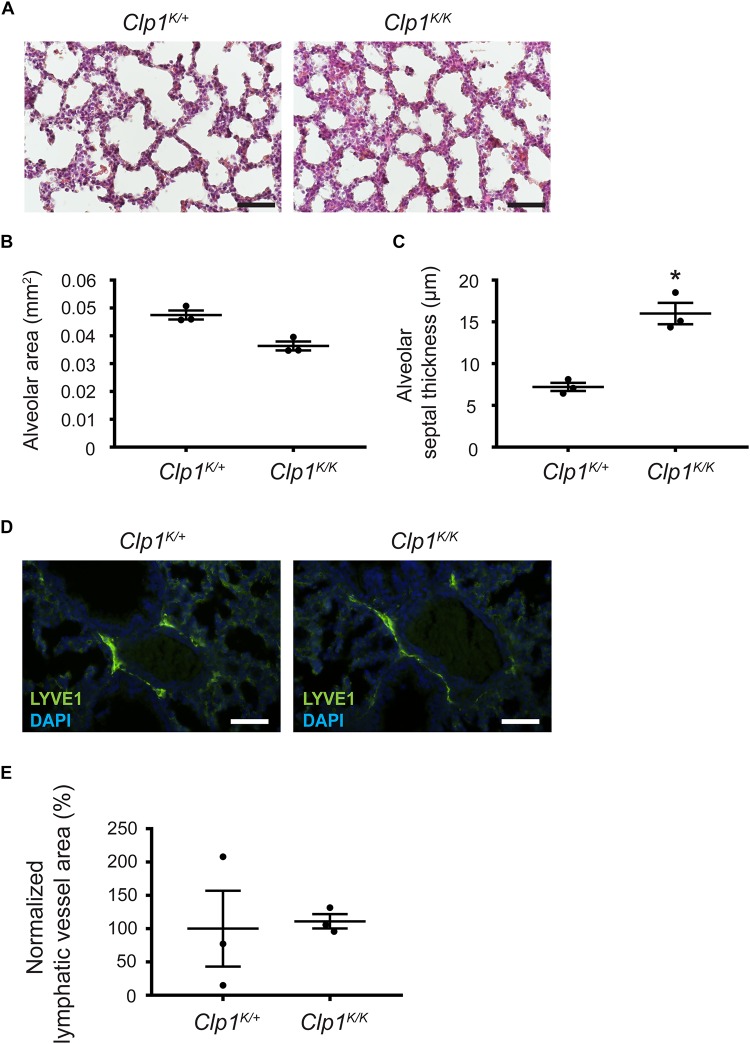
Prenatal phenotype of *Clp1*^*K/K*^ embryos on a mixed genetic background with less impaired skeletal muscle function. **(A)** Lung morphology shown by HE histology of *Clp1*^*K/+*^ and *Clp1*^*K/K*^ embryos at E18.5 on a c57Bl/6-NMRI mixed background. Representative images are shown of three embryos per group from one litter. Bars, 50 μm. **(B,C)** Quantitative data for alveolar area **(B)** and alveolar septal thickness **(C)** of embryonic lungs of *Clp1*^*K/+*^ and *Clp1*^*K/K*^ littermates on a c57Bl/6-NMRI mixed background at E18.5 represented as mean and SEM in three embryos in each group from one litter [*P* = 0.0561 for alveolar area and ^∗^*P* = 0.0281 for the septal thickness (two-tailed *t*-test)]. **(D)** Morphology of pulmonary lymphatic vessels shown by LYVE1 immunostaining in E18.5 late gestation *Clp1*^*K/+*^ and *Clp1*^*K/K*^ embryonic lungs on a c57Bl/6-NMRI mixed background. Representative images are shown of three embryos in each group from one litter. Bars, 50 μm. **(E)** Quantitative data for normalized lymphatic vessel area in *Clp1*^*K/+*^ and *Clp1*^*K/K*^ embryos at E18.5 are shown as mean and SEM of three embryos in each group from one litter [*P* = 0.8776 (two-tailed *t*-test)].

Thereafter, we characterized the lung morphology of *Clp1*^*K/K*^ embryos on the c57Bl/6-NMRI mixed genetic background at E18.5 before the air inflation of the embryonic lungs. The results indicate that the alveolar septum is thickened, but the alveolar area is not significantly reduced in late gestation *Clp1*^*K/K*^ embryos compared to the controls on a c57Bl/6-NMRI mixed genetic background with less impaired skeletal muscle function (^∗^*P* = 0.0281 for septal thickness and *P* = 0.0561 for alveolar area) (Figures 7A–C). In addition, the pulmonary lymphatics are less dilated in the late gestation *Clp1*^*K/K*^ embryos with less impaired skeletal muscle function compared to the controls on a mixed genetic background (*P* = 0.8776) [111.04 ± 10.64% normalized to the controls (mean and SEM)] (Figures 7D,E) than in *Clp1*^*K/K*^ embryos at E18.5 on the pure c57Bl/6 genetic background (^∗∗^*P* = 0.0073) [471.49 ± 69.3% normalized to the controls (mean and SEM)] (Figure 4).

These results suggest a trend indicating the correlation between the severity of the impairment of the skeletal muscle function and the dilation of pulmonary lymphatics in the developing lung.

## Discussion

In our study we used *Clp1*^*K/K*^ CLP1 kinase-dead mice which lose the innervation of skeletal muscle from E16.5 onward (Hanada et al., 2013). Our experiments confirmed the findings of the prior report (Hanada et al., 2013) demonstrating that *Clp1*^*K/K*^ newborns show impaired motor function resulting in fatal respiratory failure with cyanosis after birth indicated by the reduced skeletal muscle activity and reduced viability of *Clp1*^*K/K*^ newborns (Figure 1 and Supplementary Movie S1). Importantly, it has been reported that breathing-like episodes of the late gestation embryo correlate well with the motor functions and breathing activity of the newborn after birth, but the frequency of FBMs is less *in utero* than the frequency of the breathing after birth (Niblock et al., 2020). Therefore, our findings indicate that the *Clp1*^*K/K*^ genetic model serves as an excellent tool to study the possible role of FBMs on pulmonary development *in utero*, which periodic movements are present during late gestation.

Fetal breathing movements during late gestation have been documented not only in large animals and humans, but also in mice. In early studies premature Cesarean sections followed by plethysmography, and monitoring breathing-like movements in reduced preparations were performed (Viemari et al., 2003; Thoby-Brisson et al., 2005). Later the embryos one by one were externalized for imaging studies (Kleven and Ronca, 2009). A recent report imaging non-externalized embryos *in utero* demonstrated the presence of FBMs in wild-type unanesthetized pregnant mice by ultrasound, and described that FBMs can show sporadic, clustered, or rhythmic patterns in mice, and there is a tendency of their increase with embryonic age (Niblock et al., 2020). The authors also emphasized that detecting FBMs *in utero* in genetic mouse models has great limitations. The anesthesia of the pregnant female, reduced preparation, externalization of the embryos, or other mechanical manipulation may influence the FBMs. In connection, Brown et al. (2006) were not able to detect FBMs in mouse embryos probably due to the anesthesia. The best way to monitor FBMs is to perform imaging in the abdomen of the female without externalization of the embryos and uterus but in this case it is difficult or impossible to determine that which body part belongs to an individual embryo especially in larger litters (Niblock et al., 2020; and data not shown). Externalization of the uterus allows the precise identification of each embryo for FBM measurements by *in vivo* ultrasound and corresponding genotyping. Limitations of this method include mechanical disturbance of utero-placental circulation and a drop in body temperature. Therefore, only a limited time frame is available to observe FBMs of the embryos in this setting, while it is possible an extended period of time (more than 10 min) is needed to effectively document periodic FBMs in the case of each embryo. It has also been shown as another difficulty that FBMs are primarily present during REM sleep in sheep and cease before delivery (Dawes et al., 1972; Greer et al., 2006). Despite these significant limitations we observed FBMs in *Clp1*^+/+^ and *Clp1*^*K/+*^ control embryos at E18.5 [2.67 ± 1.12 FBMs in 2 min (mean and SEM); five out of six control embryos performed FBMs], and no FBM was detectable in 1 *Clp1*^*K/K*^ embryo with normal heart activity in one litter (Supplementary Movies S2, S3). Of note, we also had two litters at E18.5 showing no or minimal FBM activity in most of the late gestation embryos (data not shown). Further studies will be needed to develop imaging techniques to more effectively monitor FBMs in mouse genetic models.

We found that the expression of molecular and cellular markers of lung development is not altered in *Clp1*^*K/K*^ embryos using a number of developmental markers including alveolar type II and I cell, Club (Clara) cell, mesenchymal cell, and vascular cell markers (Figure 2 and Supplementary Table S1). These findings are in accordance with a previous report, in which the expression of surfactant protein C and A in late gestation *Clp1*^*K/K*^ embryos was analyzed (Hanada et al., 2013). Another prior study, in which the authors aspirated the amniotic fluid to study the possible role of the mechanical forces on lung development indicated that amniotic fluid inhalation influences alveolar type I cell differentiation, whereas FGF10-mediated ERK1/2 signaling induces a protrusive structure in some cells, which process protects from flattening to specify alveolar type II fate (Li et al., 2018). In connection to this report, it is important to note that the leakage or drainage of the amniotic fluid may affect not only FBMs but also the volume and function of the organs. Therefore, this model provides important results about the possible role of mechanical forces and fluid volumes on organ development, but they have great limitations to reveal the physiological role of FBMs.

Importantly, our studies revealed that the alveolar septa are thicker and the alveolar area is reduced in late gestation *Clp1*^*K/K*^ embryos before air inflation suggesting that mechanical forces including FBMs influence the expansion of the developing lung (Figure 3). Former studies also indicated the defect in lung expansion in models using transection of the spinal cord above the phrenic nucleus or the section of the phrenic nerve, and concluded the presence of lung hypoplasia, while a part of the results indicated that the cellularity represented by the total DNA content of the developing lung is not affected (Fewell et al., 1981; Liggins et al., 1981; Bamford et al., 1992). Of note, the surgical approaches in these former studies manipulating the spinal cord or phrenic nerve are heroic and may influence a number of other physiological processes in addition to FBMs.

It is known that mechanical forces influence the growth rate of the embryo and the skeletal system from the early embryonic period (Felsenthal and Zelzer, 2017). Therefore, it should be noted that other reports which use genetic models in which embryos are paralyzed from the early embryonic period to study the possible role of FBMs described severe defects of the whole skeleton. These changes are represented in the smaller size and reduced growth rate of the embryo, indicating the great limitations of these experimental systems (Sandler et al., 1994; Tseng et al., 2000). Importantly, the growth rate of the *Clp1*^*K/K*^ was not reduced in our studies, which is a great advantage of our experimental system (Figures 1A–D).

In addition, the *Clp1*^*K/K*^ genotype does not influence molecular and cellular lung development (Figure 2 and Supplementary Table S1). Lung hypoplasia was also not present in late gestation *Clp1*^*K/K*^ embryos showing normal DNA content and dry weights of the embryonic lungs (Figures 3D,E). This is in contrast to former studies which concluded the presence of lung hypoplasia in experiments trying to define the possible role of mechanical forces on lung development, but these findings also suggest the great limitation of the former studies using surgical approaches, modulating general growth rate of the embryo and influencing fluid volumes as discussed above.

Our prior studies indicated that parallel mechanisms prepare the developing lung for inflation at birth (Jakus et al., 2014). In addition to surfactant, prenatal lymphatic function also plays an important role to prepare the developing lung for inflation at birth (Jakus et al., 2014; Aspelund et al., 2016). Our former study using lymphatic-deficient genetic models demonstrated the importance of prenatal lymphatic function as a regulator of lung inflation at birth by inducing the expansion and increasing the compliance of the developing lung in preparation for the first breath (Jakus et al., 2014; Aspelund et al., 2016). Our current study revealed that the prenatal phenotype of the developing lung in late gestation *Clp1*^*K/K*^ embryos is very similar with thickened alveolar septa and reduced alveolar area to the phenotype that was reported in lymphatic-deficient animals (Jakus et al., 2014). Importantly, pulmonary lymphatics are present in the embryonic *Clp1*^*K/K*^ lung, but they are markedly dilated (Figure 4). Moreover, the prenatal pulmonary lymphatic function is reduced in *Clp1*^*K/K*^ embryos (Figure 5). We also detected a trend indicating the correlation between the severity of the impairment of the skeletal muscle function in *Clp1*^*K/K*^ embryos and the dilation of pulmonary lymphatics in the developing lung on a mixed genetic background (Figures 6, 7). Our results have revealed that *Clp1*^*K/K*^ mice show impaired motor functions including FBMs, and late gestation *Clp1*^*K/K*^ embryos display reduced prenatal lymphatic function and impaired lung expansion represented as thickened alveolar septa and reduced alveolar area in preparation of the developing lung for inflation at birth.

Here we found that the skeletal muscle dysfunction is less severe in *Clp1*^*K/K*^ mice on the c57Bl/6-NMRI mixed genetic background than it is on the pure c57Bl/6 genetic background. Of note, Hanada et al. (2013) also reported the most severe motor neuron loss and paralysis of *Clp1*^*K/K*^ embryos on the c57Bl/6 genetic background. No surviving Clp1^*K/K*^ newborn was reported on the c57Bl/6 genetic background in their study. It would be an interesting future project to reveal the molecular mechanism why the phenotype affecting the skeletal muscles is less severe on the c57Bl/6-NMRI mixed genetic background. Here we wanted to demonstrate that there is a trend, which indicates the correlation between the severity of the impairment of the skeletal muscle function and the dilation of pulmonary lymphatics in the developing lung.

In connection, our recent study revealed the lack of smooth muscle coverage of the collecting pulmonary lymphatics in contrast to other collecting lymphatic vessels in the body (Tammela and Alitalo, 2010; Aspelund et al., 2016; Petrova and Koh, 2018; Outtz Reed et al., 2019), implying that changes in pressure and respiratory movements may be a driver of lymphatic function in the lung as opposed to contraction of the vessel itself. This suggests that lymphatic function, both pre- and postnatally may be dependent on breathing movements. One intriguing interpretation of the data presented here is that the mechanism how FBMs regulate the expansion of the developing lung is to stimulate the pulmonary lymphatic function before the air inflation of the lung and the presence of postnatal respiratory activity. It would be an important next step in the project to test possible pharmacological approaches (e.g. caffeine) in large animal models to stimulate FBMs during late gestation, which treatment may prevent neonatal respiratory failure in preterm infants.

The *Clp1*^*K/K*^ late gestation embryos exhibit a progressive loss of motor neurons and skeletal muscle activity from E16.5 onward during late gestation, and it is not possible to rescue the motor neuron loss at or after birth. We think that it is as good or better model for studying the impact of FBMs on lung development than the other approaches (leakage or drainage of the amniotic fluid, performing heroic surgery, paralysis starting at an early developmental stage, etc.). To provide further experimental proof has great limitations. The first possible way would be to rescue the lymphatic function in late gestation *Clp1*^*K/K*^ embryos. To this end, it would be necessary to generate rhythmic contractions of the chest, basically mimicking breathing like movements *in utero*. This would be a possible way how the mechanical forces generated around the pulmonary lymphatic vessels lacking smooth muscle coverage could rescue the lymphatic function in these paralyzed late gestation *Clp1*^*K/K*^ embryos, but it is not a feasible experiment. The second possible additional way would be to use a model in which motor activity and FBMs are impaired during late gestation before birth, but the embryos show normal respiration after birth. We do not know about a model like this. The third possible way would be to set up a mouse intensive care unit, in which it would be possible to treat an anesthetized, mechanically ventilated pregnant wild-type mouse during late gestation with skeletal muscle relaxant drugs. This experiment would also be very challenging. In all possible models it would be useful to monitor FBMs and lymphatic function in each embryo in parallel *in utero*, but it is not feasible to perform this experiment in mouse embryos *in vivo*, especially for an extended period of time.

Collectively, the characterization of the development of the embryonic lung before air inflation in this study revealed that the alveolar septa are thicker, and the alveolar area is reduced in late gestation *Clp1*^*K/K*^ embryos, which model shows impaired skeletal muscle function including FBMs, while the molecular lung development is not affected. Importantly, pulmonary lymphatic vessels appear to be dilated and the prenatal pulmonary lymphatic function is reduced in *Clp1*^*K/K*^ embryos. Thus, our results have revealed that *Clp1*^*K/K*^ mice show impaired motor functions including FBMs, and late gestation *Clp1*^*K/K*^ embryos display reduced prenatal lymphatic function and impaired lung expansion represented as thickened alveolar septa and reduced alveolar area in preparation of the developing lung for inflation at birth. These findings suggest a possible mechanism that FBMs, similarly to breathing movements after birth, stimulate prenatal lymphatic function in pulmonary collecting lymphatics lacking smooth muscle coverage to prepare the developing lung for inflation and gas exchange at birth. Moreover, these results raise the possibility that stimulating FBMs during late gestation might be an effective way to reduce the risk of the development of neonatal respiratory failure.

## Data Availability Statement

All datasets generated for this study are included in the article/Supplementary Material.

## Ethics Statement

All animal experiments were approved by the Animal Experimentation Review Board of the Semmelweis University and the Government Office for Pest County (Hungary).

## Author Contributions

KS-A, MK, and ZJ designed the work. KS-A, DS, GK, JA, and ZJ performed the experiments and analyzed the data. GB and ZG designed and performed *in utero* ultrasound imaging. MK and ZJ initiated the study. JP provided experimental tools and gave scientific advice. KS-A and ZJ interpreted the results and wrote the manuscript with the help of the other authors. ZJ supervised the project.

## Conflict of Interest

The authors declare that the research was conducted in the absence of any commercial or financial relationships that could be construed as a potential conflict of interest.

## Supplementary Material

TABLE S1 Quantification of lung developmental and structural markers in *Clp1*^+/+^ and *Clp1*^*K/K*^ embryos at E18.5. Quantification of structures or cell types in immunofluorescent images. Cell counts are presented as cell/mm^2^ (CC10, SPC, PDPN, PDGFRα, NG2, Desmin, and Vimentin). Vascular structure counts (PDGFRβ, α-SMA, PECAM1, VEGFR3, and PROX1/LYVE1) are shown as mean and SEM. PDPN, Desmin, PDGFRα, NG2, and Vimentin are quantified on an area of 100 μm ^∗^ 100 μm. CC10, SPC, LYVE1 and PROX1, PDGFRβ, and VEGFR3 are quantified on a field of view of images made with a 40× objective. PECAM and α-SMA are quantified on a field of view of images made with a 20× objective. No data showed significant difference between the two groups (mean ± SEM, two-tailed *t*-test).

MOVIE S1 Representative movie of *Clp1*^+/+^ and *Clp1*^*K/K*^ newborn mice 20 min after birth.

MOVIE S2 Representative ultrasound movie of late gestation *Clp1*^*K/+*^ control embryo at E18.5. Note the respiration of the pregnant female.

MOVIE S3 Representative ultrasound movie of late gestation *Clp1*^*K/K*^ embryo at E18.5. Note the respiration of the pregnant female.

## Abbreviations

α-SMA: α-smooth muscle actin
CC10: club cell 10 (Clara cell 10)
DAPI: 4 ′, 6-diamidino-2-phenylindole
E: embryonic day
FBMs: fetal breathing movements
HE: hematoxylin eosin
LEC: lymphatic endothelial cell
LYVE1: lymphatic vessel endothelial hyaluronan receptor 1
NG2: neuron–glial antigen 2
P: postnatal day
PBS: phosphate-buffered saline
PDGFR α: platelet-derived growth factor receptor α
PDGFR β: platelet-derived growth factor receptor β
PDPN: podoplanin
PECAM1: platelet endothelial cell adhesion molecule 1
PROX1: prospero homeobox 1
RhD: phodamine-dextran
SPC: prosurfactant protein C
VEGFR3: vascular endothelial growth factor receptor 3.
