# Supplementary figures and images for: Reduced Prenatal Pulmonary Lymphatic Function Is Observed in Clp1K/K Embryos With Impaired Motor Functions Including Fetal Breathing Movements in Preparation of the Developing Lung for Inflation at Birth
Source: Front Bioeng Biotechnol. 2020 Mar 6;8:136. doi: 10.3389/fbioe.2020.00136 (PMC7067749; doi:10.3389/fbioe.2020.00136)

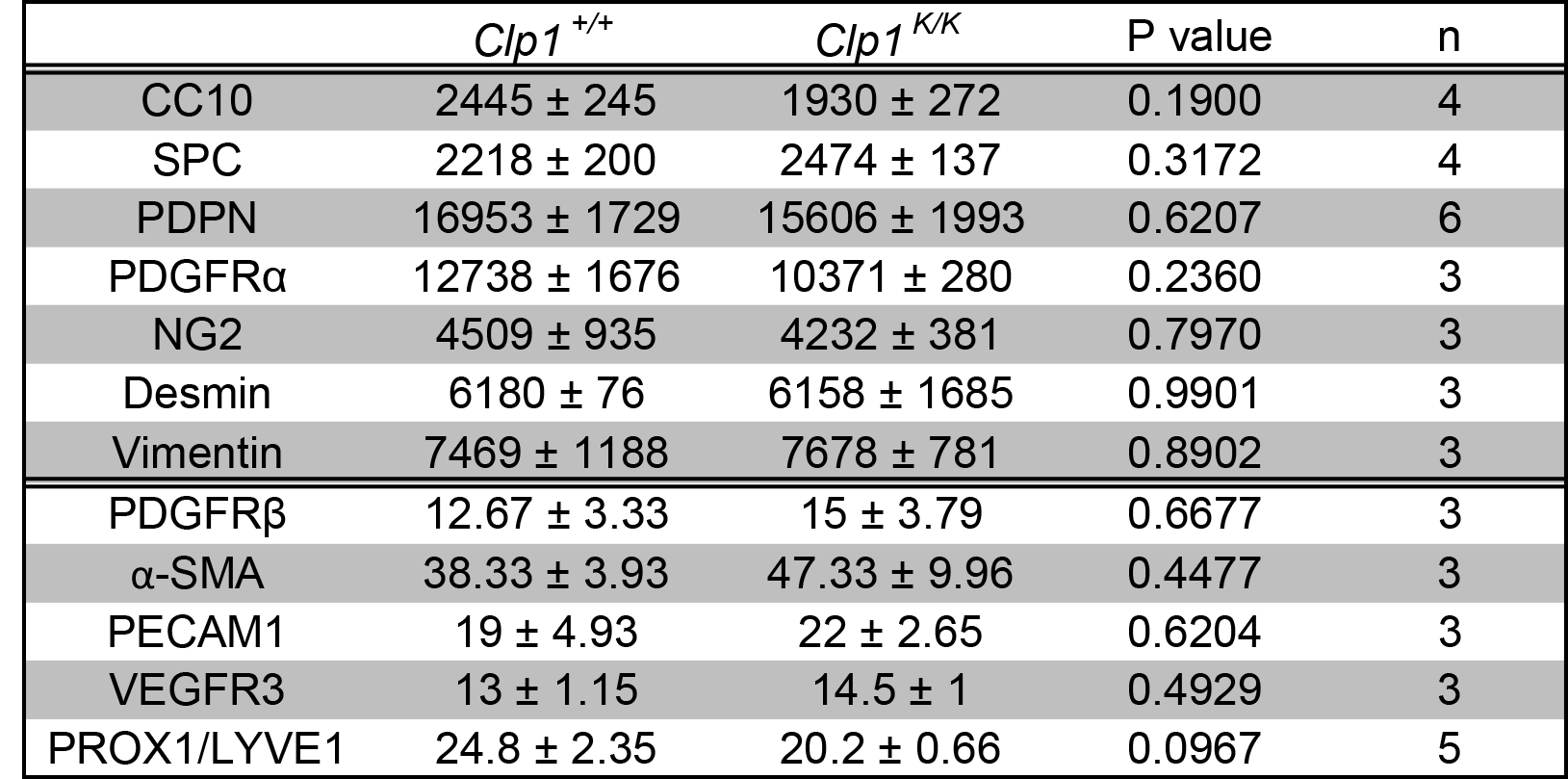

Supplement: TABLE S1 — Quantification of lung developmental and structural markers in Clp1+/+ and Clp1K/K embryos at E18.5. Quantification of structures or cell types in immunofluorescent images. Cell counts are presented as cell/mm2 (CC10, SPC, PDPN, PDGFRα, NG2, Desmin, and Vimentin). Vascular structure counts (PDGFRβ, α-SMA, PECAM1, VEGFR3, and PROX1/LYVE1) are shown as mean and SEM. PDPN, Desmin, PDGFRα, NG2, and Vimentin are quantified on an area of 100 μm ∗ 100 μm. CC10, SPC, LYVE1 and PROX1, PDGFRβ, and VEGFR3 are quantified on a field of view of images made with a 40× objective. PECAM and α-SMA are quantified on a field of view of images made with a 20× objective. No data showed significant difference between the two groups (mean ± SEM, two-tailed t-test). [file Data_Sheet_1.zip › Table 1.tif]
